# Reference genome of the long-jawed orb-weaver, *Tetragnatha versicolor* (Araneae: Tetragnathidae)

**DOI:** 10.1093/jhered/esad013

**Published:** 2023-04-12

**Authors:** Seira A Adams, Natalie R Graham, Anna J Holmquist, Monica M Sheffer, Emma C Steigerwald, Ruta Sahasrabudhe, Oanh Nguyen, Eric Beraut, Colin Fairbairn, Samuel Sacco, William Seligmann, Merly Escalona, H Bradley Shaffer, Erin Toffelmier, Rosemary G Gillespie

**Affiliations:** Department of Environmental Science, Policy and Management, University of California, Berkeley, CA, United States; Center for Population Biology, University of California, Davis, CA, United States; Department of Evolution and Ecology, University of California, Davis, CA, United States; Department of Environmental Science, Policy and Management, University of California, Berkeley, CA, United States; Department of Environmental Science, Policy and Management, University of California, Berkeley, CA, United States; Department of Biology, University of Washington, Seattle, WA, United States; eScience Institute, University of Washington, Seattle, WA, United States; Department of Environmental Science, Policy and Management, University of California, Berkeley, CA, United States; Museum of Vertebrate Zoology, University of California, Berkeley, CA, United States; DNA Technologies and Expression Analysis Core Laboratory, Genome Center, University of California, Davis, CA, United States; DNA Technologies and Expression Analysis Core Laboratory, Genome Center, University of California, Davis, CA, United States; Department of Ecology and Evolutionary Biology, University of California, Santa Cruz, CA, United States; Department of Ecology and Evolutionary Biology, University of California, Santa Cruz, CA, United States; Department of Ecology and Evolutionary Biology, University of California, Santa Cruz, CA, United States; Department of Ecology and Evolutionary Biology, University of California, Santa Cruz, CA, United States; Department of Biomolecular Engineering, University of California, Santa Cruz, CA, United States; Department of Ecology and Evolutionary Biology, University of California, Los Angeles, CA, United States; La Kretz Center for California Conservation Science, Institute for Environment and Sustainability, University of California, Los Angeles, CA, United States; Department of Ecology and Evolutionary Biology, University of California, Los Angeles, CA, United States; La Kretz Center for California Conservation Science, Institute for Environment and Sustainability, University of California, Los Angeles, CA, United States; Department of Environmental Science, Policy and Management, University of California, Berkeley, CA, United States

**Keywords:** California Conservation Genomics Project, CCGP, arachnid, spider genome

## Abstract

Climate-driven changes in hydrological regimes are of global importance and are particularly significant in riparian ecosystems. Riparian ecosystems in California provide refuge to many native and vulnerable species within a xeric landscape. California *Tetragnatha* spiders play a key role in riparian ecosystems, serving as a link between terrestrial and aquatic elements. Their tight reliance on water paired with the widespread distributions of many species make them ideal candidates to better understand the relative role of waterways versus geographic distance in shaping the population structure of riparian species. To assist in better understanding population structure, we constructed a reference genome assembly for *Tetragnatha versicolor* using long-read sequencing, scaffolded with proximity ligation Omni-C data. The near-chromosome-level assembly is comprised of 174 scaffolds spanning 1.06 Gb pairs, with a scaffold N50 of 64.1 Mb pairs and BUSCO completeness of 97.6%. This reference genome will facilitate future study of *T. versicolor* population structure associated with the rapidly changing environment of California.

## Introduction

Climate change has had, and will continue to have, a significant effect on the world’s natural ecosystems and consequently on the services that these systems provide to humanity (IPCC 2022). However, it is clear that successful local and global mitigation efforts can help vulnerable ecosystems persist through forthcoming changes (Greenwood *et al*. 2016; Owen 2020). Riparian ecosystems have been shown to be one of the most vulnerable to climate change and anthropogenic disturbances due to the acute dependency of these systems on water availability, flow rate and direction, as well as temperature and habitat modifications (Capon *et al*. 2013). Riparian ecosystems in California are in a particularly precarious position because they exist in a heavily populated and fundamentally xeric landscape where the effects of anthropogenic disturbance and climate change are widespread (Seavy *et al*. 2009; Rohde *et al*. 2021). These riparian ecosystems provide refuge to many native and imperiled species, both aquatic and terrestrial, and therefore will play a key role in mediating the adaptability of California biodiversity in response to climate change (Capon *et al*. 2013; Bogan *et al*. 2019). Without effective conservation and management strategies, these ecosystems are at risk. Critical to any management approach is an understanding of the connectivity within and between the various rivers and subwatersheds.

Members of the spider family Tetragnathidae (Araneae), the long-jawed spiders, and in particular the genus *Tetragnatha*, are prevalent in riparian habitats, characterized by their orb-webs constructed over bodies of water. They play an integral part in the riparian ecosystem as both predator and prey, providing a trophic connection between terrestrial and aquatic ecosystems (Akamatsu *et al*. 2004). Species are often widely distributed but depend on bodies of water as they are sensitive to desiccation (Gillespie 1987; Adams 2022). In California, there are 6 species of *Tetragnatha* spiders: *T. versicolor*, *T. laboriosa*, *T. pallescens*, *T. nitens*, *T. elongata*, and *T. guatemalensis*. *T. versicolor* Walckenaer, 1841 is the most abundant and widespread species, and is found across a wide range of ecoregions (Levi 1981), though always in strict association with water. This narrow niche requirement in addition to its widespread nature makes *T. versicolor* an ideal taxon to study how populations are structured, whether by the waterways themselves or by geographic distance. Insights into how the species is being affected by recent shifts in hydrological regimes connected to anthropogenic change require more detailed genomic assessment. To this end, we constructed a reference genome assembly of *T. versicolor* as part of the California Conservation Genomics Project (CCGP, Shaffer *et al*. 2022) to facilitate an investigation of the population structure of California *Tetragnatha* species and its response to climate change in California.

## Materials and methods

### Biological materials

**Fig. 1.**
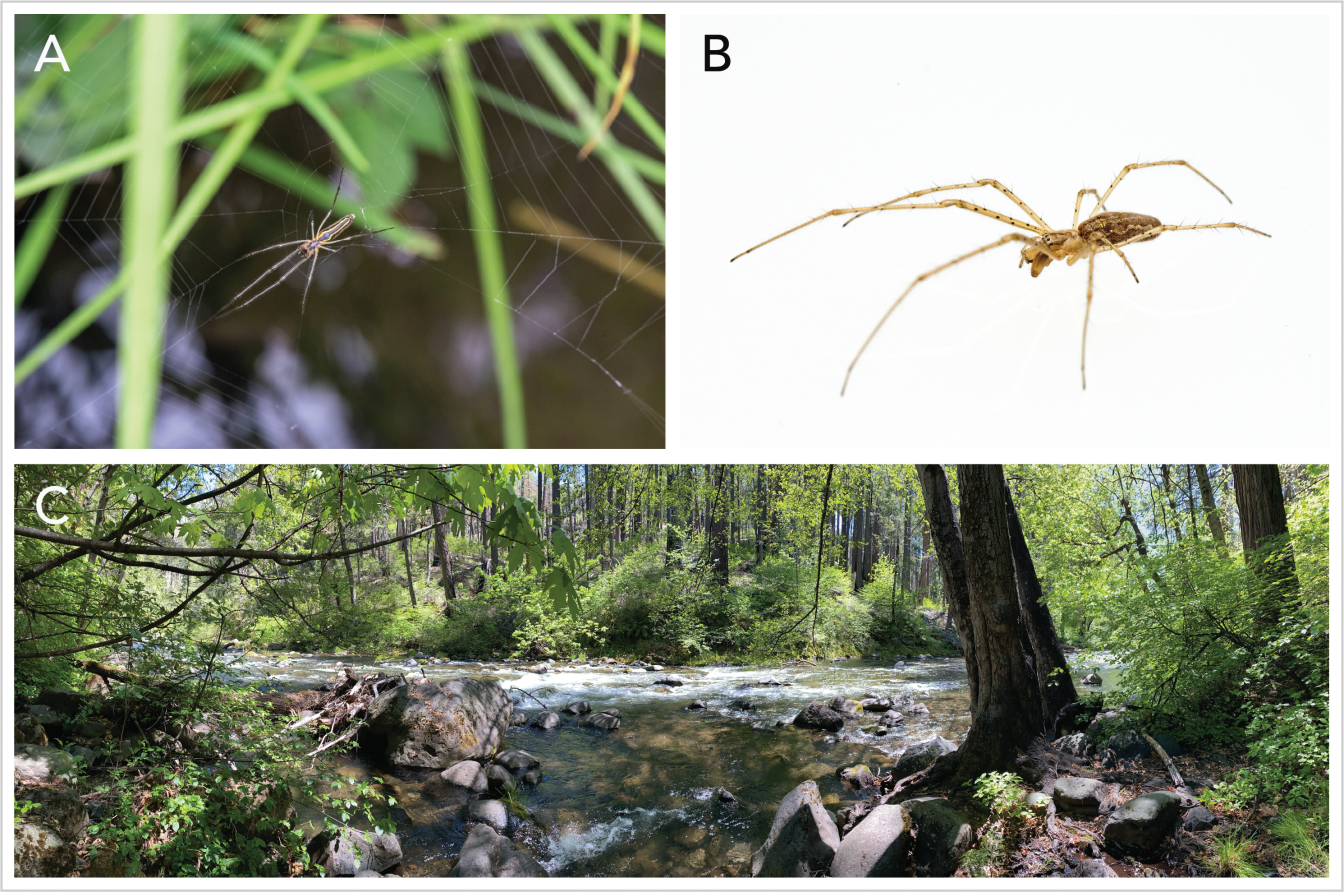
A) Ventral view of a *Tetragnatha versicolor* spider on a web over water. B) A dorsal view of a *T. versicolor* female. C) A representative riparian habitat of *T. versicolor* in Tehama County, California.

Adult female *T. versicolor* (Fig. 1) were hand collected from plants on the banks of the Eel River in Angelo Coast Range Reserve in Branscomb, California (39.72114°N, 123.648963°W) on 2 October 2020. Specimens were kept alive until DNA extraction. One individual (CCGP_27_UCD_003, SAMN29044170) was used for HiFi library preparation and sequencing while another individual (CCGP_27_UCSC_010; SAMN29044171) was used for Omni-C library preparation and sequencing. Female specimens were chosen since females have the X_1_X_1_X_2_X_2_ sex chromosome system and they are generally larger than the male spiders that have the X_1_X_2_0 sex chromosome system.

### Nucleic acid library preparations and DNA sequencing

#### DNA extraction.

One entire female spider (CCGP_27_UCD_003) was flash frozen in liquid nitrogen (LN_2_) and homogenized by grinding in a mortar and pestle in the presence of LN_2_. Homogenized tissue was lysed with 2 ml of lysis buffer containing 100 mM NaCl, 10 mM Tris-HCL-pH 8.0, 25 mM EDTA, 0.5% SDS, and 100 µg/ml proteinase K overnight at room temperature. The lysate was treated with 20 µg/ml RNAse at 37 °C for 30 min and cleaned with equal volumes of phenol/chloroform using phase-lock gels (Quantabio, Beverly, Massachusetts; Cat # 2302830). The DNA was precipitated by adding 0.4× volume of 5 M ammonium acetate and 3× volume of ice-cold ethanol. The DNA pellet was washed twice with 70% ethanol and resuspended in an elution buffer (10 mM Tris, pH 8.0). genetic DNA (gDNA) purity was accessed using a NanoDrop ND-1000 spectrophotometer, which returned a 260/280 ratio of 1.9 and 260/230 of 2.0. DNA yield (12 µg total) was quantified using Qbit 2.0 Fluorometer (Thermo Fisher Scientific, Waltham, Massachusetts). Integrity of the high molecular weight (HMW) gDNA was verified on a Femto pulse system (Agilent Technologies, Santa Clara, California) where 60% of the DNA was found in fragments above 50 kb and 50% of the DNA was found in fragments above 120 kb.

#### HiFi library preparation and sequencing.

A HiFi SMRTbell library was constructed using the SMRTbell Express Template Prep Kit v2.0 (Pacific Biosciences—PacBio, Menlo Park, California, Cat. #100-938-900) according to the manufacturer’s instructions. HMW gDNA was sheared to a target DNA size distribution between 15 and 20 kb. The sheared gDNA was concentrated using 0.45× of AMPure PB beads (PacBio, Cat. #100-265-900) for the removal of single-strand overhangs at 37 °C for 15 min, followed by further enzymatic steps of DNA damage repair at 37 °C for 30 min, end repair and A-tailing at 20 °C for 10 min and 65 °C for 30 min, ligation of overhang adapter v3 at 20 °C for 60 min and 65 °C for 10 min to inactivate the ligase, and nuclease treatment at 37 °C for 1 h. The SMRTbell library was purified and concentrated with 0.45× Ampure PB beads (PacBio, Cat. #100-265-900) for size selection using the BluePippin/PippinHT system (Sage Science, Beverly, Massachusetts; Cat. #BLF7510/HPE7510) to collect fragments greater than 7 to 9 kb. The 15 to 20 kb average HiFi SMRTbell library was sequenced at University of California Davis DNA Technologies Core (Davis, California) using 2 8M SMRT cells, Sequel II sequencing chemistry 2.0, and 30-h movies each on a PacBio Sequel II sequencer.

#### Omni-C library preparation and sequencing.

The Omni-C library was prepared using the Dovetail Omni-C Kit (Dovetail Genomics, Scotts Valley, California) according to the manufacturer’s protocol with slight modifications. First, specimen tissue (using whole individual spider CCGP_27_UCSC_010) was thoroughly ground with a mortar and pestle while cooled with LN_2_. Subsequently, chromatin was fixed in place in the nucleus. The suspended chromatin solution was then passed through 100 and 40 μm cell strainers to remove large debris. Fixed chromatin was digested under various conditions of DNase I until a suitable fragment length distribution of DNA molecules was obtained. Chromatin ends were repaired and ligated to a biotinylated bridge adapter followed by proximity ligation of adapter containing ends. After proximity ligation, crosslinks were reversed and the DNA purified from proteins. Purified DNA was treated to remove biotin that was not internal to ligated fragments, and an NGS library was generated using an NEB Ultra II DNA Library Prep kit (New England Biolabs, Ipswich, Massachusetts) with an Illumina compatible y-adaptor. Biotin-containing fragments were then captured using streptavidin beads, and the post-capture product was split into 2 replicates prior to PCR enrichment to preserve library complexity with each replicate receiving unique dual indices. The libraries were sequenced at the Vincent J. Coates Genomics Sequencing Lab (Berkeley, California) on an Illumina NovaSeq platform (Illumina, San Diego, California) to generate approximately 100 million 2 × 150 bp read pairs per GB of genome size.

### Nuclear genome assembly

We assembled the genome of the long-jawed spider following the CCGP assembly pipeline Version 4.0, as outlined in Table 1 which lists the tools and non-default parameters used in the assembly. The pipeline uses PacBio HiFi reads and Omni-C data to produce high quality and highly contiguous genome assemblies while minimizing manual curation. We removed remnant adapter sequences from the PacBio HiFi dataset using HiFiAdapterFilt (Sim *et al*. 2022) and obtained the initial dual or partially phased diploid assembly (http://lh3.github.io/2021/10/10/introducing-dual-assembly) using HiFiasm (Cheng *et al*. 2022) with the filtered PacBio HiFi reads and the Omni-C dataset. We tagged output haplotype 1 as the primary assembly, and output haplotype 2 as the alternate assembly. We identified sequences corresponding to haplotypic duplications, contig overlaps and repeats on the primary assembly with purge_dups (Guan *et al*. 2020) and transferred them to the alternate assembly. We aligned the Omni-C data to both assemblies following the Arima Genomics Mapping Pipeline (https://github.com/ArimaGenomics/mapping_pipeline) and then scaffolded both assemblies with SALSA (Ghurye *et al*. 2017, 2019).

**Table 1.** Assembly pipeline and software used.

Assembly	Software and options^a^	Version
Filtering PacBio HiFi adapters	HiFiAdapterFilt	Commit 64d1c7b
K-mer counting	Meryl (k = 21)	1
Estimation of genome size and heterozygosity	GenomeScope	2
*De novo assembly (contiging)*	HiFiasm (Hi-C Mode, –primary, output p_ctg.hap1, p_ctg.hap2)	0.16.1-r375
Remove low-coverage, duplicated contigs	purge_dups	1.2.5
Scaffolding		
Omni-C Scaffolding	SALSA (-DNASE, -i 20, -p yes)	2
Gap closing	YAGCloser (-mins 2 -f 20 -mcc 2 -prt 0.25 -eft 0.2 -pld 0.2)	Commit0e34c3b
Omni-C Contact map generation		
Short-read alignment	BWA-MEM (-5SP)	0.7.17-r1188
SAM/BAM processing	Samtools	1.11
SAM/BAM filtering	Pairtools	0.3.0
Pairs indexing	Pairix	0.3.7
Matrix generation	Cooler	0.8.10
Matrix balancing	hicExplorer (hicCorrectmatrix correct --filterThreshold -2 4)	3.6
Contact map visualization	HiGlass	2.1.11
	PretextMap	0.1.4
	PretextView	0.1.5
	PretextSnapshot	0.0.3
Organelle assembly		
Mitogenome assembly	MitoHiFi (-r, -p 50, -o 1)	2 Commitc06ed3e
Genome quality assessment		
Basic assembly metrics	QUAST (--est-ref-size)	5.0.2
Assembly completeness	BUSCO (-m geno, -l arthropoda)	5.0.0
	Merqury	2020-01-29
Contamination screening		
Local alignment tool	BLAST+	2.1
General contamination screening	BlobToolKit	2.3.3

Software citations are listed in the text.

^a^Options detailed for non-default parameters.

We generated Omni-C contact maps for both assemblies by aligning the Omni-C data with BWA-MEM (Li 2013), identified ligation junctions, generated Omni-C pairs using pairtools (Goloborodko *et al*. 2018), generated a multi-resolution Omni-C matrix with cooler (Abdennur and Mirny 2020), and balanced it with hicExplorer (Ramírez *et al*. 2018). We used HiGlass (Kerpedjiev *et al*. 2018) and the PretextSuite (https://github.com/wtsi-hpag/PretextView; https://github.com/wtsi-hpag/PretextMap; https://github.com/wtsi-hpag/PretextSnapshot) to visualize the contact maps and checked the contact maps for major misassemblies. If we identified a strong off-diagonal signal and a lack of signal in the consecutive genomic region in the proximity of a join that was made by the scaffolder, we marked the join. Afterwards, all marked joins were dissolved by cutting the scaffolds at the coordinates of the joins. After this process, no further manual joins were made. Some of the remaining gaps (joins) were closed using the PacBio HiFi reads and YAGCloser (https://github.com/merlyescalona/yagcloser). We then checked for contamination using the BlobToolKit Framework (Challis *et al*. 2020). Finally, we trimmed remnants of sequence adaptors and mitochondrial contamination identified during NCBI contamination screening.

### Mitochondrial genome assembly

We assembled the mitochondrial genome of the long-jawed spider from the PacBio HiFi reads using the reference-guided pipeline MitoHiFi (https://github.com/marcelauliano/MitoHiFi; Allio *et al*. 2020). The mitochondrial sequence of *T. nitens* (NCBI:NC_028068.1; Wang *et al*. 2016) was used as the starting reference sequence. After completion of the nuclear genome, we searched for matches of the resulting mitochondrial assembly sequence in the nuclear genome assembly using BLAST+ (Camacho *et al*. 2009) and filtered out contigs and scaffolds from the nuclear genome with a percentage of sequence identity >99% and size smaller than the mitochondrial assembly sequence.

### Genome size estimation and quality assessment

We generated k-mer counts from the PacBio HiFi reads using meryl (https://github.com/marbl/meryl). The k-mer database was then used in GenomeScope2.0 (Ranallo-Benavidez *et al*. 2020) to estimate genome features including genome size, heterozygosity, and repeat content. To obtain general contiguity metrics, we ran QUAST (Gurevich *et al*. 2013). To evaluate genome quality and completeness we used BUSCO (Manni *et al*. 2021) with the arthropoda ortholog database (arthropoda_odb10) which contains 1,013 genes. Assessment of base level accuracy (QV) and k-mer completeness was performed using the previously generated meryl database and merqury (Rhie *et al*. 2020). We further estimated genome assembly accuracy via BUSCO gene set frameshift analysis using the pipeline described in Korlach *et al*. (2017). Measurements of the size of the phased blocks is based on the size of the contigs generated by HiFiasm on HiC mode. We follow the quality metric nomenclature established by Rhie *et al*. (2021), with the genome quality code x.y.P.Q.C, where, x = log10[contig NG50]; y = log10[scaffold NG50]; *P* = log10[phased block NG50]; Q = Phred base accuracy QV (quality value); C = % genome represented by the first “n” scaffolds, following a known karyotype of 2n = 24 inferred from the congeneric *T. maxillosa* (The Animal Chromosome Count database—V1.0.0; https://cromanpa94.github.io/ACC/). Quality metrics for the notation were calculated on the primary assembly.

## Results

**Fig. 2.**
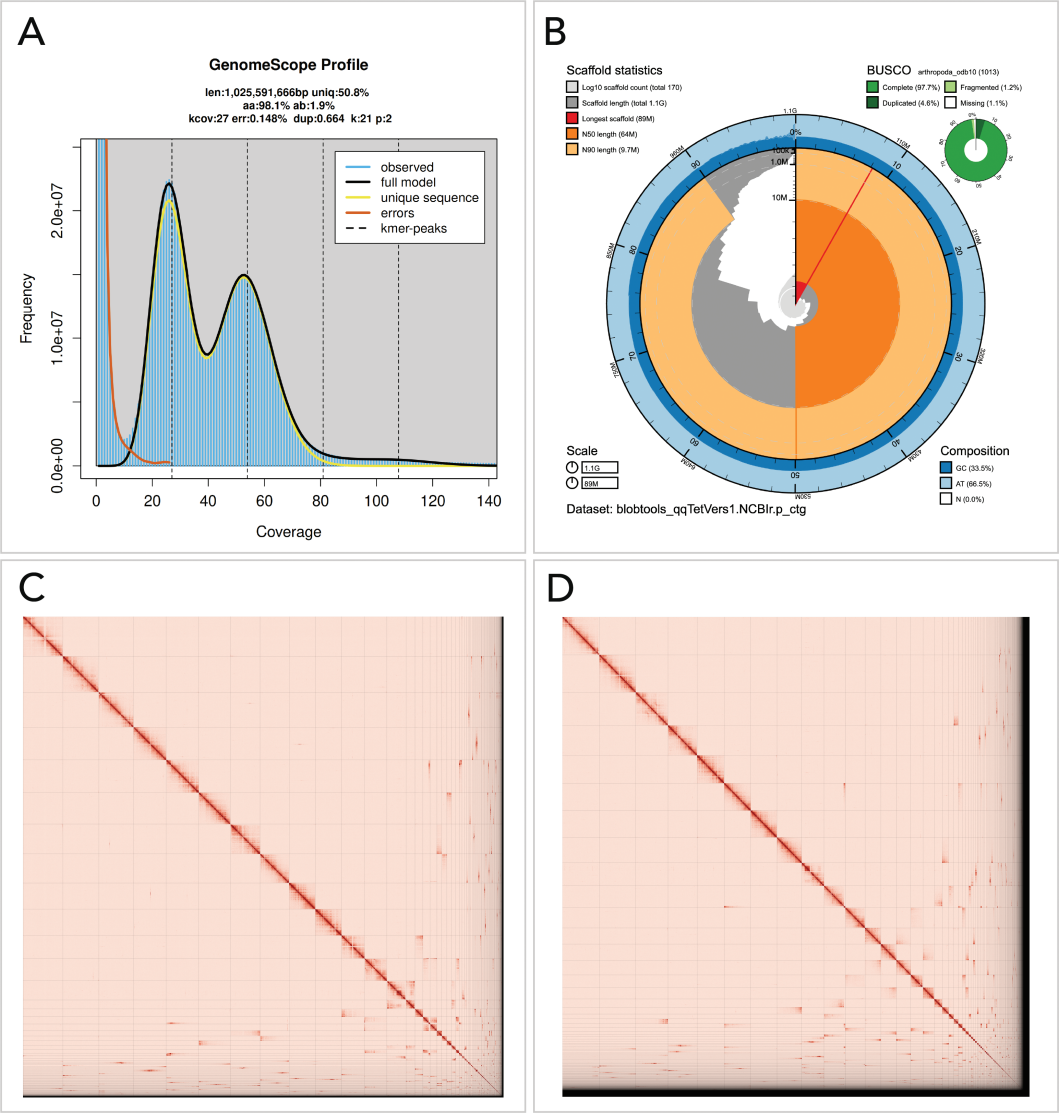
Visual overview of *Tetragnatha versicolor* genome assembly metrics. A) K-mer spectrum output generated from PacBio HiFi data without adapters using GenomeScope2.0. The bimodal pattern observed corresponds to a diploid genome and the k-mer profile matches that of high heterozygosity. K-mers at lower coverage and high frequency correspond to differences between haplotypes, whereas the higher coverage and low frequency k-mers correspond to the similarities between haplotypes. B) BlobToolKit Snail plot showing a graphical representation of the quality metrics presented in Table 2 for the *T. versicolor* primary assembly (qqTetVers1.0.p). The plot circle represents the full size of the assembly. From the inside-out, the central plot covers length-related metrics. The red line represents the size of the longest scaffold; all other scaffolds are arranged in size-order moving clockwise around the plot and drawn in gray starting from the outside of the central plot. Dark and light orange arcs show the scaffold N50 and scaffold N90 values. The central light gray spiral shows the cumulative scaffold count with a white line at each order of magnitude. White regions in this area reflect the proportion of Ns in the assembly; the dark vs. light blue area around it shows mean, maximum and minimum GC vs AT content at 0.1% intervals (Challis *et al*. 2020). C and D) HiC Contact maps for the primary (2C) and alternate (2D) genome assembly generated with PretextSnapshot. Hi-C contact maps translate proximity of genomic regions in 3-D space to contiguous linear organization. Each cell in the contact map corresponds to sequencing data supporting the linkage (or join) between 2 of such regions. Scaffolds are separated by black lines and higher density of the lines may correspond to higher levels of fragmentation.

The Omni-C and PacBio HiFi sequencing libraries generated 91.4 million read pairs and 3.29 million reads, respectively. The latter yielded 55.64-fold coverage (N50 read length 17,735 bp; minimum read length 49 bp; mean read length 17,343 bp; maximum read length of 61,245 bp) based on the Genomescope 2.0 genome size estimation of 1.09 Gb. Based on PacBio HiFi reads, we estimated 0.148% sequencing error rate and 1.9% nucleotide heterozygosity rate. The k-mer spectrum based on PacBio HiFi reads show a bimodal distribution with 2 major peaks at 27- and 55-fold coverage, where peaks correspond to homozygous and heterozygous states of a diploid species (Fig. 2A). The distribution presented in this k-mer spectrum supports that of a high heterozygosity profile.

The final assembly (qqTetVers1) consists of 2 pseudo haplotypes, primary and alternate, with both genome sizes similar to the estimated value from Genomescope2.0 (Fig. 2A). The primary assembly consists of 174 scaffolds spanning 1.06 Gb with contig N50 of 9 Mb, scaffold N50 of 64.1 Mb, longest contig of 38.5 Mb, and largest scaffold of 88.6 Mb. GC content composition for the primary assembly is 33.5% and AT content 66.5%. The alternate assembly is similar, although it consists of 539 scaffolds spanning 1.03 Gb with contig N50 of 7.6 Mb, scaffold N50 of 55.1 Mb, largest contig 24 Mb, and largest scaffold of 82.5 Mb. GC and AT content composition for the alternate assembly are similar to the primary, with 33.6% for GC content and 66.4% for AT content. Detailed assembly statistics are reported in Table 2, and graphically for the primary assembly in Fig. 2B (see Supplementary Figure 1 for the alternate assembly). The primary assembly has a BUSCO completeness score of 97.7% using the Arthropoda gene set, a per-base quality (QV) of 66.35, a k-mer completeness of 76.05, and a frameshift indel QV of 52.6; while the alternate assembly has a BUSCO completeness score of 96.1% using the same gene set, a per-base quality (QV) of 65.08, a k-mer completeness of 74.02, and a frameshift indel QV of 55.48.

**Table 2.** Sequencing and assembly statistics, and accession numbers.

Bio Projects & Vouchers	CCGP NCBI BioProject			PRJNA720569			
	Genera NCBI BioProject			PRJNA765871			
	Species NCBI BioProject			PRJNA851111			
	NCBI BioSample			SAMN29044170, SAMN29044171			
	Specimen identification			CCGP_RG_IND1, TVERS_CCGP_IND2			
	NCBI Genome accessions			**Primary**		**Alternate**	
	Assembly accession			JANDEH000000000		JANDEI000000000	
	Genome sequences			GCA_024610705.1		GCA_024610695.1	
Genome ­Sequence	PacBio HiFi reads		Run	1 PACBIO_SMRT (Sequel II) run: 3.3M spots, 57.1G bases, 38.9Gb			
			Accession	SRX16742514			
	Omni-C Illumina reads		Run	2 ILLUMINA (Illumina NovaSeq 6000) runs: 91.4M spots, 27.6G bases, 9.4Gb			
			Accession	SRX16742515, SRX16742516			
Genome Assembly Quality Metrics	Assembly identifier (Quality code^a^)			qqTetVers1(6.7.P6.Q65.C91)			
	HiFi Read coverage^b^			55.64X			
				**Primary**		**Alternate**	
	Number of contigs			301		661	
	Contig N50 (bp)			9,004,227		7,638,610	
	Contig NG50^b^			9,481,185		8,296,675	
	Longest Contigs			38,560,849		24,030,258	
	Number of scaffolds			174		539	
	Scaffold N50			64,139,176		55,124,716	
	Scaffold NG50^b^			66,476,450		55,124,716	
	Largest scaffold			88,624,587		82,515,128	
	Size of final assembly			1,068,089,209		1,039,239,443	
	Phased block NG50^b^			9,561,114		8,343,110	
	Gaps per Gbp (# Gaps)			120 (128)		117 (122)	
	Indel QV (Frame shift)			52.60453018		55.48708725	
	Base pair QV			66.3573		65.0859	
				Full assembly = 65.6839			
	k-mer completeness			76.055		74.0291	
				Full assembly = 98.8437			
	BUSCO completeness (arthropoda) n=1013		**C**	**S**	**D**	**F**	**M**
		P^c^	97.70%	93.10%	4.60%	1.20%	1.10%
		A^c^	96.10%	92.10%	4.00%	1.80%	2.10%
	Organelles		1 complete mitochondrial sequence			CM045180.1	

^a^Assembly quality code x.y.P.Q.C derived notation, from (Rhie *et al*. 2021). x = log10[contig NG50]; y = log10[scaffold NG50]; P = log10 [phased block NG50]; Q = Phred base accuracy QV (Quality value); C = % genome represented by the first “n” scaffolds, following a known karyotype of 2n = 24 from another species from the same genus *Tetragnatha maxillosa*. Quality code for all the assembly denoted by primary assembly (qqTetVers1.0.p).

^b^Read coverage and NGx statistics have been calculated based on the estimated genome size of 1.025 Gb.

^c^(P)rimary and (A)lternate assembly values.

We identified 21 misassemblies, 9 on the primary and 12 on the alternate, and broke the corresponding joins made by SALSA. We were able to close a total of 14 gaps, 9 on the primary assembly and 5 on the alternate. Finally, we filtered out 3 contigs, 1 from the primary and 2 from the alternate assembly, corresponding to mitochondrial contamination. No further contigs were removed. The Omni-C contact maps shows that both assemblies are highly contiguous (Fig. 2C and D). We have deposited both assemblies on NCBI (see Table 2 and Data Availability for details).

The assembled mitochondrial genome is 14,426 bp in length, with base composition of A = 39.95%, C = 17.11%, G = 10.38%, T = 32.56%, and includes 20 unique transfer RNAs and 12 protein coding genes.

## Discussion

Of the 16 spider genome assemblies that have been published at time of writing (Supplementary Table 1), *T. versicolor* has the smallest assembled genome size, at 1.060 Gb. Its congeneric and close relative, *T. kauaensis,* has a very similar genome assembly size of 1.085 Gb (Cerca *et al*. 2021). The remaining spider genome assemblies range in size from 1.222 Gb (*Latrodectus hesperus*, Thomas *et al*. 2020) to 6.255 Gb (*Acanthoscurria geniculata*, Sanggaard *et al*. 2014; Supplementary Table 1). Our sequence assembly generated a reference genome with contig N50 of 9 Mb. The data clustered into 13 chromosome-candidate scaffolds (Fig. 2), which is consistent with a karyotype of 2n = 26 chromosomes (Pajpach 2018). Therefore, our assembly is comparable to the 3 chromosome-level spider genome assemblies available at the time of writing: *Argiope bruennichi* (Sheffer *et al*. 2021), *Trichonephila antipodiana* (Fan *et al.* 2021), and *Dysdera silvatica* (Escuer *et al*. 2022).

Using this *T. versicolor* reference genome, we are conducting more detailed genomic analyses of *Tetragnatha* spiders using genome resequencing as part of the CCGP. These data will allow us to investigate the importance of water proximity, drought stress, and habitat connectivity in structuring populations across ecoregions, as well as enabling the study of adaptive responses to climate and other anthropogenic changes. Additionally, we will be able to assess demographic patterns and ask how habit conversion, aridification, and alteration in waterways through time has structured *Tetragnatha* populations. These data, in combination with other freshwater aquatic taxa included in the CCGP, will help build our overall understanding of the genetic structure and patterns of gene flow among riverine taxa that is central to management and conservation efforts (Fiedler *et al*. 2022; Shaffer *et al*. 2022), as well as filling in a key position in our understanding of the phylogenetic diversity of California taxa (Toffelmier *et al*. 2022). We will also use the *T. versicolor* genome to understand venom evolution, and the possible role of venoms in mate recognition (Zobel-Thropp *et al*. 2018). Moreover, comparison with the closely related Hawaiian *T. kauaiensis* will provide insights into the loss of web-building behaviors and the loss of an obligate association with water that characterize many of the Hawaiian *Tetragnatha* including *T. kauaiensis* (Berger *et al*. 2021).

Studying genomic structure and response to drought in *Tetragnatha* spiders will also provide valuable information on the health of riparian biological communities. *Tetragnatha* play crucial roles in riparian ecosystems as the primary predators of insects emerging from aquatic larval stages (Okuma 1968; Barrion and Litsinger 1984) and as a source of prey for other predatory taxa, notably birds (Gunnarsson and Wiklander 2015). Both their integral nature in riparian communities and their sensitivity to changes in water availability make them important bioindicators (Reyes-Maldonado *et al*. 2017). Understanding the genetic diversity, demographic structure, and population connectivity of *Tetragnatha* across habitats experiencing different levels of environmental change will therefore provide valuable insight into the response of the ecosystem to climate change.

## Supplementary Material

esad013_suppl_Supplementary_Figure_S1

esad013_suppl_Supplementary_Materials

## Data Availability

Data generated for this study are available under NCBI BioProject PRJNA851111. Raw sequencing data for sample CCGP_RG_IND1 and TVERS_CCGP_IND2 (NCBI BioSamples SAMN29044170 and SAMN29044171) are deposited in the NCBI Short Read Archive (SRA) under SRR20722016 for PacBio HiFi sequencing data, and SRR20722014-5 for the Omni-C Illumina sequencing data. GenBank accessions for both primary and alternate assemblies are JANDEH000000000 and JANDEI000000000; and for genome sequences GCA_024610705.1 and GCA_024610695.1. The GenBank organelle genome assembly for the mitochondrial genome is CM045180.1. Assembly scripts and other data for the analyses presented can be found at the following GitHub repository: www.github.com/ccgproject/ccgp_assembly

## References

[CIT0001] Abdennur N , Mirny LA. Cooler: scalable storage for Hi-C data and other genomically labeled arrays. Bioinformatics. 2020;36(1):311–316. doi: 10.1093/bioinformatics/btz540 31290943 PMC8205516

[CIT0002] Adams, SA. Chemical cues in species recognition and reproductive isolation of *Tetragnatha* spiders (Araneae: Tetragnathidae) [Doctoral dissertation]. UC Berkeley; 2022.

[CIT0003] Akamatsu F , Toda H, Okino T. Food source of riparian spiders analyzed by using stable isotope ratios. Ecol Res. 2004;19(6):655–662.

[CIT0004] Allio R , Schomaker-Bastos A, Romiguier J, Prosdocimi F, Nabholz B, Delsuc F. MitoFinder: efficient automated large-scale extraction of mitogenomic data in target enrichment phylogenomics. Mol Ecol Resour. 2020;20(4):892–905. doi: 10.1111/1755-0998.13160 32243090 PMC7497042

[CIT0005] Barrion AT , Litsinger JA. The spider fauna of Philippine rice agroecosystems. II. Wetland. Philipp Entomol. 1984;6(1):11–37.

[CIT0006] Berger CA , Brewer MS, Kono N, Nakamura H, Arakawa K, Kennedy SR, Wood HM, Adams SA, Gillespie RG. Shifts in morphology, gene expression, and selection underlie web loss in Hawaiian *Tetragnatha* spiders. BMC Ecol Evol. 2021;21(1):1–17. 33752590 10.1186/s12862-021-01779-9 PMC7983290

[CIT0007] Bogan MT , Leidy RA, Neuhaus L, Hernandez CJ, Carlson SM. Biodiversity value of remnant pools in an intermittent stream during the great California drought. Aquat Conserv Mar Freshwater Ecosyst. 2019;29(6):976–989. doi: 10.1002/aqc.3109

[CIT0008] Camacho C , Coulouris G, Avagyan V, Ma N, Papadopoulos J, Bealer K, Madden TL. BLAST+: architecture and applications. BMC Bioinf. 2009;10(1):1–9. 10.1186/1471-2105-10-421 PMC2803857 20003500

[CIT0009] Capon SJ , Chambers LE, Mac Nally R, Naiman RJ, Davies P, Marshall N, Pittock J, Reid M, Capon T, Douglas M, et al. Riparian ecosystems in the 21st century: hotspots for climate change adaptation? Ecosystems. 2013;16(3):359–381. doi: 10.1007/s10021-013-9656-1

[CIT0010] Cerca J , Armstrong EE, Vizueta J, Fernández R, Dimitrov D, Petersen B, Prost S, Rozas J, Petrov D, Gillespie RG. The *Tetragnatha kauaiensis* genome sheds light on the origins of genomic novelty in spiders. Genome Biol Evol. 2021;13(12):evab262. doi: 10.1093/gbe/evab262 34849853 PMC8693713

[CIT0011] Challis R , Richards E, Rajan J, Cochrane G, Blaxter M. BlobToolKit–interactive quality assessment of genome assemblies. G3 Genes Genomes Genet. 2020;10(4):1361–1374. 10.1534/g3.119.400908 PMC7144090 32071071

[CIT0012] Cheng H , Jarvis ED, Fedrigo O, Koepfli K-P, Urban L, Gemmell NJ, Li H. Robust haplotype-resolved assembly of diploid individuals without parental data. ArXiv Preprint ArXiv:2109.04785. 2022, doi: 10.1038/s41587-022-01261-x. PMC9464699 35332338

[CIT0013] Escuer P , Pisarenco VA, Fernández-Ruiz AA, Vizueta J, Sánchez-Herrero JF, Arnedo MA, Sánchez-Gracia A, Rozas J. The chromosome-scale assembly of the Canary Islands endemic spider *Dysdera silvatica* (Arachnida, Araneae) sheds light on the origin and genome structure of chemoreceptor gene families in chelicerates. Mol Ecol Resour. 2022;22(1):375–390. 34268885 10.1111/1755-0998.13471

[CIT0014] Fan Z , Yuan T, Liu P, Wang LY, Jin JF, Zang F, Zhang ZS. A chromosome-level genome of the spider Trichonephila antipodiana reveals the genetic basis of its polyphagy and evidence of an ancient whole-genome duplication event. Gigascience. 2021;10(3):giab016. doi: 10.1093/gigascience/giab016 33739402 PMC7976613

[CIT0015] Fiedler PL , Erickson B, Esgro M, Gold M, Hull JM, Norris J, Shapiro B, Westphal M, Toffelmier E, Shaffer HB. Seizing the moment: the opportunity and relevance of the California Conservation Genomics Project to state and federal conservation policy. J Hered. 2022;113(6):589–596. doi: 10.1093/jhered/esac046 36136001 PMC9709969

[CIT0016] Ghurye J , Pop M, Koren S, Bickhart D, Chin C-S. Scaffolding of long read assemblies using long range contact information. BMC Genomics. 2017;18(1):527. doi: 10.1186/s12864-017-3879-z 28701198 PMC5508778

[CIT0017] Ghurye J , Rhie A, Walenz BP, Schmitt A, Selvaraj S, Pop M, Phillippy AM, Koren S. Integrating Hi-C links with assembly graphs for chromosome-scale assembly. PLoS Comput Biol. 2019;15(8):e1007273. doi: 10.1371/journal.pcbi.1007273 31433799 PMC6719893

[CIT0018] Gillespie RG. The mechanism of habitat selection in the long-jawed orb-weaving spider *Tetragnatha elongata* (Araneae, Tetragnathidae). J Arachn. 1987;15(1):81–90.

[CIT0019] Goloborodko A , Abdennur N, Venev S, Brandao HB, Fudenberg G. mirnylab/pairtools: V0.2.0. Zenodo; 2018. doi:10.5281/zenodo.1490831

[CIT0020] Greenwood O , Mossman HL, Suggitt AJ, Curtis RJ, Maclean IMD. Using *in situ* management to conserve biodiversity under climate change. J Appl Ecol. 2016;53(3):885–894. doi: 10.1111/1365-2664.12602 27609987 PMC4991270

[CIT0021] Guan D , McCarthy SA, Wood J, Howe K, Wang Y, Durbin R. Identifying and removing haplotypic duplication in primary genome assemblies. Bioinformatics. 2020;36(9):2896–2898. doi: 10.1093/bioinformatics/btaa025 31971576 PMC7203741

[CIT0022] Gunnarsson B , Wiklander K. Foraging mode of spiders affects risk of predation by birds. Biol J Linn Soc. 2015;115(1):58–68. doi: 10.1111/bij.12489

[CIT0023] Gurevich A , Saveliev V, Vyahhi N, Tesler G. QUAST: quality assessment tool for genome assemblies. Bioinformatics. 2013;29(8):1072–1075. doi: 10.1093/bioinformatics/btt086 23422339 PMC3624806

[CIT0024] IPCC. Climate change 2022: impacts, adaptation and vulnerability. Contribution of working group II to the sixth assessment report of the intergovernmental panel on climate change. Cambridge University Press; 2022.

[CIT0025] Kerpedjiev P , Abdennur N, Lekschas F, McCallum C, Dinkla K, Strobelt H, Luber JM, Ouellette SB, Azhir A, Kumar N, et al. HiGlass: web-based visual exploration and analysis of genome interaction maps. Genome Biol. 2018;19(1):125. doi: 10.1186/s13059-018-1486-1 30143029 PMC6109259

[CIT0026] Korlach J , Gedman G, Kingan SB, Chin C-S, Howard JT, Audet J-N, Cantin L, Jarvis ED. De novo PacBio long-read and phased avian genome assemblies correct and add to reference genes generated with intermediate and short reads. GigaScience. 2017;6(10):gix085. doi: 10.1093/gigascience/gix085 PMC5632298 29020750

[CIT0027] Levi H. W. American orb-weaver genera Dolichognatha and *Tetragnatha* north of Mexico (Araneae: Araneidae, Tetragnathinae). Bulletin of the Museum of Comparative Zoology; 1981;149:271–318.

[CIT0028] Li H. Aligning sequence reads, clone sequences and assembly contigs with BWA-MEM. ArXiv Preprint ArXiv:1303.3997. 2013. doi: 10.48550/arXiv.1303.3997.

[CIT0029] Manni M , Berkeley MR, Seppey M, Simão FA, Zdobnov EM. BUSCO update: novel and streamlined workflows along with broader and deeper phylogenetic coverage for scoring of eukaryotic, prokaryotic, and viral genomes. Mol Biol Evol. 2021;38(10):4647–4654. doi: 10.1093/molbev/msab199 34320186 PMC8476166

[CIT0030] Okuma C. Preliminary survey on the spider-fauna of the paddy fields in Thailand. Mushi 1968;42(Pars 8):89–118.

[CIT0031] Owen G. What makes climate change adaptation effective? A systematic review of the literature. Global Environ Change. 2020;62:102071.

[CIT0032] Pajpach, F. (2018). Karyotype evolution of the family Araneidae. Charles University.

[CIT0033] Ramírez F , Bhardwaj V, Arrigoni L, Lam KC, Grüning BA, Villaveces J, Habermann B, Akhtar A, Manke T. High-resolution TADs reveal DNA sequences underlying genome organization in flies. Nat Commun. 2018;9(1):1–15. 29335486 10.1038/s41467-017-02525-w PMC5768762

[CIT0034] Ranallo-Benavidez TR , Jaron KS, Schatz MC. GenomeScope 2.0 and Smudgeplot for reference-free profiling of polyploid genomes. Nat Commun. 2020;11(1):1–10. 32188846 10.1038/s41467-020-14998-3 PMC7080791

[CIT0035] Reyes-Maldonado R , Sánchez-Ruiz JA, Kelly SP. Riparian spider communities as indicators of stream ecosystem condition in the Río Piedras watershed of Puerto Rico. Actual Biol. 2017;39(107):58–65.

[CIT0036] Rhie A , McCarthy SA, Fedrigo O, Damas J, Formenti G, Koren S, Uliano-Silva M, Chow W, Fungtammasan A, Kim J, et al. Towards complete and error-free genome assemblies of all vertebrate species. Nature. 2021;592(7856):737–746. 33911273 10.1038/s41586-021-03451-0 PMC8081667

[CIT0037] Rhie A , Walenz BP, Koren S, Phillippy AM. Merqury: reference-free quality, completeness, and phasing assessment for genome assemblies. Genome Biol. 2020;21(1):1–27. 10.1186/s13059-020-02134-9 PMC7488777 32928274

[CIT0038] Rohde MM , Stella JC, Roberts DA, Singer MB. Groundwater dependence of riparian woodlands and the disrupting effect of anthropogenically altered streamflow. Proc Natl Acad Sci USA. 2021;118(25):e2026453118. 34161277 10.1073/pnas.2026453118 PMC8237578

[CIT0039] Sanggaard KW , Bechsgaard JS, Fang X, Duan J, Dyrlund TF, Gupta V, Jiang X, Cheng L, Fan D, Feng Y, et al. Spider genomes provide insight into composition and evolution of venom and silk. Nat Commun. 2014;5(1):Article 1. doi:10.1038/ncomms4765 PMC4273655 24801114

[CIT0040] Seavy NE , Gardali T, Golet GH, Griggs FT, Howell CA, Kelsey R, Small SL, Viers JH, Weigand JF. Why climate change makes riparian restoration more important than ever: recommendations for practice and research. Ecol Restor. 2009;27(3):330–338.

[CIT0041] Shaffer HB , Toffelmier E, Corbett-Detig RB, Escalona M, Erickson B, Fiedler P, Gold M, Harrigan RJ, Hodges S, Luckau TK, et al. Landscape genomics to enable conservation actions: the California Conservation Genomics Project. J Hered. 2022;113:577–588. 10.1093/jhered/esac020 35395669

[CIT0042] Sheffer MM , Hoppe A, Krehenwinkel H, Uhl G, Kuss AW, Jensen L, Jensen C, Gillespie RG, Hoff KJ, Prost S. Chromosome-level ­reference genome of the European wasp spider *Argiope bruennichi*: a resource for studies on range expansion and evolutionary adaptation. GigaScience. 2021;10(1):giaa148. 33410470 10.1093/gigascience/giaa148 PMC7788392

[CIT0043] Sim SB , Corpuz RL, Simmonds TJ, Geib SM. HiFiAdapterFilt, a memory efficient read processing pipeline, prevents occurrence ofadapter sequence in PacBio HiFi reads and their negative impacts on genome assembly. BMC Genomics. 2022;23(1):1–7. 35193521 10.1186/s12864-022-08375-1 PMC8864876

[CIT0044] Thomas GW , Dohmen E, Hughes DS, Murali SC, Poelchau M, Glastad K, Anstead CA, Ayoub NA, Batterham P, Bellair M. Gene content evolution in the arthropods. Genome Biol. 2020;21(1):1–14. 10.1186/s13059-019-1925-7 PMC6977273 31969194

[CIT0045] Toffelmier E , Beninde J, Shaffer HB. The phylogeny of California, and how it informs setting multi-species conservation priorities. J Hered. 2022;113(6):597–603. doi: 10.1093/jhered/esac045 36048626 PMC9709974

[CIT0046] Wang Z-L , Li C, Fang W-Y, Yu X-P. The complete mitochondrial genome of two *Tetragnatha* spiders (Araneae: Tetragnathidae): severe truncation of tRNAs and novel gene rearrangements in Araneae. Int J Biol Sci. 2016;12(1):109–119. 26722222 10.7150/ijbs.12358 PMC4679403

[CIT0047] Zobel-Thropp PA , Bulger EA, Cordes MH, Binford GJ, Gillespie RG, Brewer MS. Sexually dimorphic venom proteins in long-jawed orb-weaving spiders (*Tetragnatha*) comprise novel gene families. PeerJ. 2018;6:e4691. 29876146 10.7717/peerj.4691 PMC5985773

