# Supplementary figures and images for: Reference genome of the long-jawed orb-weaver, Tetragnatha versicolor (Araneae: Tetragnathidae)
Source: J Hered. 2023 Apr 12;114(4):395–403. doi: 10.1093/jhered/esad013 (PMC10287146; doi:10.1093/jhered/esad013)

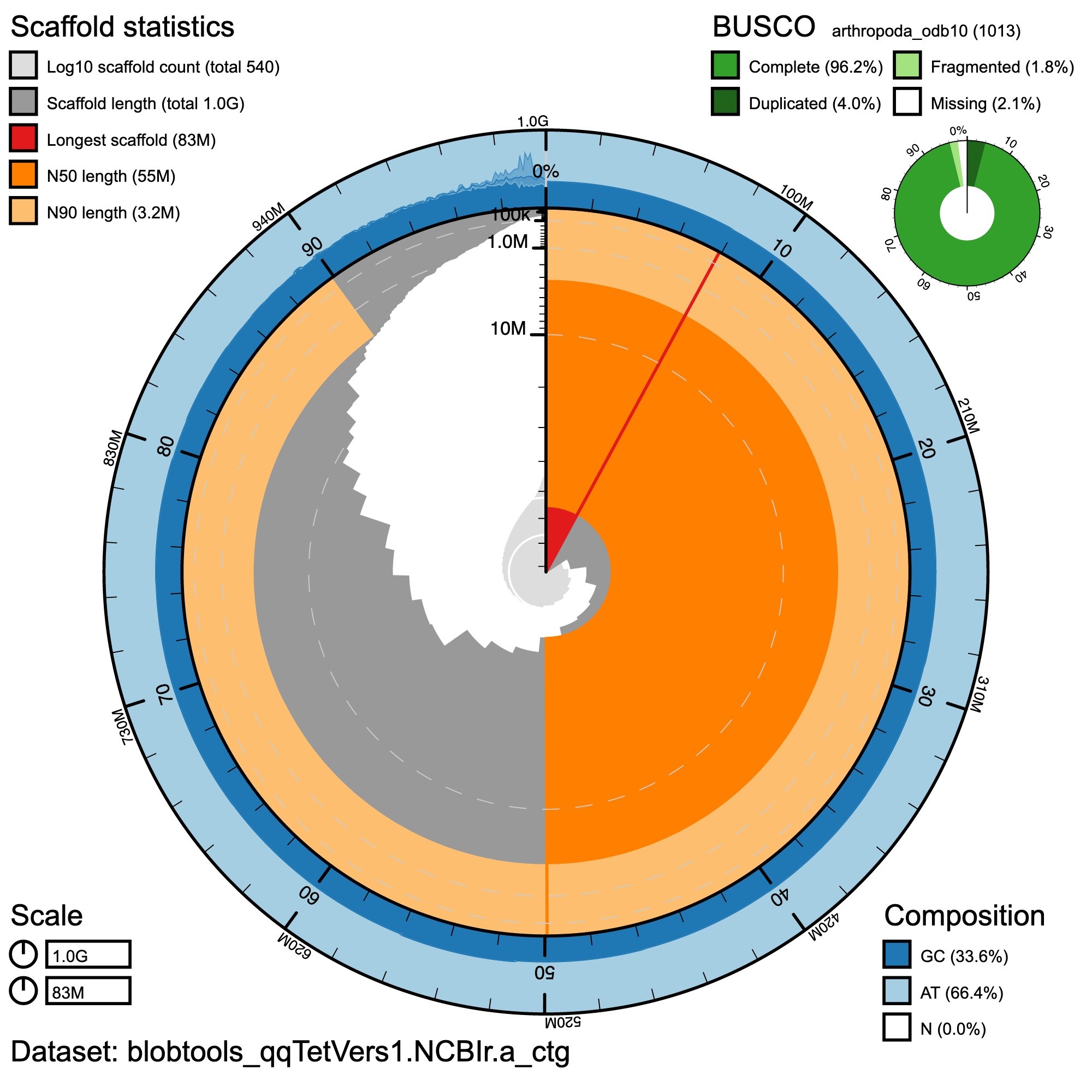

Supplement: esad013_suppl_Supplementary_Figure_S1 [file esad013_suppl_supplementary_figure_s1.jpeg]
