# Supplementary material for: Reference genome of the long-jawed orb-weaver, Tetragnatha versicolor (Araneae: Tetragnathidae)
Source: J Hered. 2023 Apr 12;114(4):395–403. doi: 10.1093/jhered/esad013 (PMC10287146; doi:10.1093/jhered/esad013)
Supplement: esad013_suppl_Supplementary_Materials [file esad013_suppl_supplementary_materials.docx]

# SUPPLEMENTARY MATERIALS

**SM Figure 1** - BlobToolKit Snail plot showing a graphical representation of the quality metrics presented in Table 2 for the *T. versicolor* alternate assembly (qqTetVers1.0.a).

**SM Table 1** - Various statistics of the whole genome of other Arachnids published to date.

| **Species** | **Genome size (bp)** | **# of contigs** | **Largest contig (bp)** | **Scaffold N50 (bp)** | **%GC** | **reference** |
| --- | --- | --- | --- | --- | --- | --- |
| *Tetragnatha versicolor* | 1,068,075,909 | 301 | 38,560,849 | 64,139,176 | 33.5 | This article |
| *Tetragnatha kauaiensis* | 1,085,571,486 | 3,925 | 10,500,000 | 2,000,000 | 33.3 | (Cerca et al., 2021) |
| *Araneus ventricosus* | 3,656,621,265 | 300,721 | 9,335,346 | 59,619 | 31.9 | (Kono et al., 2019) |
| *Argiope bruennichi* | 1,670,285,661 | 2,231 | 143,171,375 | 124,235,998 | 29.33 | (Sheffer et al., 2021) |
| *Caerostris darwini* | 1,500,000,000 | 15,733 | 3,567,188 | 440,877 | 29.9 | (Kono et al., 2021) |
| *Caerostris extrusa* | 1,420,000,000 | 21,729 | 799,240 | 98,474 | 29.6 | (Kono et al., 2021) |
| *Trichonephila clavipes* | 2,439,269,210 | 180,124 | 1,655,743 | 62,959 | 31.59 | (Babb et al., 2017) |
| *Trichonephila antipodiana* | 2,290,000,000 | 377 | 230,170,000 | 172,892,000 | 31.7 | (Fan et al., 2021) |
| *Anelosimus studiosus* | 1,929,674,296 | 638,008 | 78,878 | 5,142 | 27.83 | unpublished |
| *Latrodectus hesperus* | 1,222,317,290 | 122,909 | 677,069 | 40,091 | 27.25 | (Thomas et al., 2020) |
| *Parasteatoda tepidariorum* | 1,226,240,701 | 52,036 | 7,604,257 | 765,739 | 29.4 | (Schwager et al., 2017) |
| *Stegodyphus mimosarum* | 2,727,074,523 | 26,864 | 4,549,800 | 482,171 | 33.62 | (Sanggaard et al., 2014) |
| *Stegodyphus dumicola* | 2,550,000,000 | 16,532 | 1,740,957 | 254,130 | 33.26 | (Liu et al., 2019) |
| *Pardosa pseudoannulata* | 4,126,186,519 | 362,797 | 8,106,735 | 727,225 | 31.32 | (Yu et al., 2019) |
| *Loxosceles reclusa* | 3,262,472,151 | 143,648 | 715,838 | 63,237 | 39.38 | (Thomas et al., 2020) |
| *Dysdera silvatica* | 1,365,686,336 | 15,360 | 317,950,000 | 174,190,000 | 34.75 | (Escuer et al., 2022) |
| *Acanthoscurria geniculata* | 6,255,640,349 | 1,865,928 | 849,588 | 28,571 | 40.08 | (Sanggaard et al., 2014) |
